# Chain-mediated effects of career self-efficacy and career planning between social support and career anxiety in college soccer players

**DOI:** 10.3389/fpsyg.2025.1685011

**Published:** 2025-11-26

**Authors:** Yu-Juan Feng, Zhen-Yu Zhang, Wei Wang, Wen-Juan Zhang, Guang-Wei Che

**Affiliations:** 1 Teaching Department of Common Courses, Shandong University of Art & Design, Jinan, China; 2 Graduate School, Shandong Sport University, Jinan, China; 3 Department of Modern Handicrafts, Shandong University of Art & Design, Jinan, China; 4 Department of Physical Education, Qufu Normal University, Qufu, China; 5 Department of Rehabilitation Medicine, Shandong University of Traditional Chinese Medicine, Jinan, China

**Keywords:** university-level high-performance soccer players, social support, career anxiety, career choice self-efficacy, career planning, chain mediation

## Abstract

The purpose of this study was to investigate the factors affecting the Career Anxiety of Chinese university high-level soccer players, to construct a chain mediation model, and to provide intervention suggestions to alleviate the Career Anxiety of this group. The study used questionnaires and collected 797 valid questionnaires (404 male; 393 female), and used AMOS 21.0 and SPSS 19.0 software for data processing and model testing through hierarchical regression and structural equation modeling. The results showed that: social support had a direct negative effect on Career Anxiety; career choice self-efficacy and career planning mediated between social support and Career Anxiety, respectively; and career choice self-efficacy and career planning together constituted a significant chain mediation effect. The conclusion suggests that improving the social support level, enhancing their career choice self-efficacy and perfecting their career planning ability are the key ways to effectively intervene in Career Anxiety among college high-level soccer players.

## 1 Introduction

The employment of Chinese college students has always been a focal social issue (Erica and Harry, 2019). With the rapid development of China’s society and economy, the job market demands ever-increasing talent levels, and the employment challenges facing college students have become increasingly serious (Wang and Zhang, 2023). Within this demographic, high-performance university-level soccer players face a greater employment challenge. High-level college soccer players are soccer players recruited by the state, with the assistance of the university education platform. They are expected to surpass a certain level of soccer abilities to qualify for university entry. The purpose of recruiting these athletes is to improve the overall strength of Chinese soccer and promote more rapid development of the sport; however, actual results remain very low (Yu et al., 2019). Compared with professional soccer players, the overall competitive strength of university high-level soccer players is relatively low, while, compared with ordinary college students, the academic level of this group is also low (Gouttebarge et al., 2016). University high-level soccer players struggle to maintain a balance between training and study, but must compete with general entry college students after graduation. Therefore, unemployment among this group of students is high.

In the current fierce career market, high-level university soccer players may experience deep anxiety owing to employment pressure. This is manifested in a sense of powerlessness, inner restlessness, and mental rigidity in the face of employment. They often feel worried and panicked, and can even fear job-hunting, all of which endangers their mental health (Confectioner et al., 2021). Owing to conflicts between university study and training time, these athletes often appear to be insufficient in knowledge and employability, while also lacking sufficient professional internships and practical experience, putting them in a relatively unfavorable position in the job market. Female high-level soccer players also report poor employment outcomes and very narrow employment fields, so that women have higher levels of career anxiety than men.

Although current research on career anxiety has attracted the attention of a wide range of scholars, research on the employment anxiety of high-level college soccer players remains insufficient. Appropriate career anxiety can promote an individual’s employment skills, but excessive career anxiety can affect both physical and mental health (Bao et al., 2023). Therefore, to help university high-level soccer players to adjust their mindset, ease their anxiety, and face employment with a positive mindset, this study explores the influencing factors affecting this group’s employment anxiety and its psychological mechanism, and provides a theoretical basis for improving anxiety and ensuring adequate preparation for the job market.

Social Cognitive Career Theory (SCCT), based on social cognitive theory (Cui and Gu, 2024), considers internal factors, environmental factors, and individual behavior as the core elements, and constructs a framework for individual career choice and development, which has been widely used in the study of students’ professional education and career choices, employment, and entrepreneurship (Khyati et al., 2023; Suwaluck and Wisuwat, 2022). Social Cognitive Career Theory is built around three core concepts: self-efficacy, outcome expectancy, and career goals. This theory posits that an individual’s career expectations and development are influenced not only by their own cognition but also closely linked to environmental factors. From an environmental perspective, socioeconomic conditions—such as the economic impact of the COVID-19 pandemic—directly shape students’ career expectations and choices. Support from family or school also influences university students’ career preferences. From an individual perspective, personality traits, career adaptability, self-efficacy, and self-esteem levels can all potentially affect an individual’s career preferences and expectations. Based on SCCT, this study explores the effects of social support on career anxiety and its psychological mechanisms among Chinese university high-level soccer players.

There are currently few studies on the subject of career anxiety among college high-level soccer player-specific groups, and most career anxiety studies focus on the general college student population. Dyrbye et al. (2006) believes that the social interactions of college students and the external factors of the campus environment have a strong influence on employment stress. Tian (2012) found that the uncertainty encountered by college students in the job search process exacerbated their employment stress, and the stress generated by external factors contributed to the stage of elevated career anxiety. More social resources and social support can alleviate college students’ anxiety caused by external stressors. Wu and Zhao’s (2023) study found that increasing college students’ subjective social support was effective in alleviating their Career Anxiety. A study by Bao et al. (2020) concluded that college students’ perceived social support made them feel more meaningful in life, thus reducing Career Anxiety. Huang et al. (2025) showed that college students’ perceived social support improved their depressive symptoms and increased self-efficacy. An in-depth study by Yang (2009), on the career anxiety of Chinese third- and fourth-year college students, indicated a significant negative correlation between the social support received by college students and their level of career anxiety. Shi and Ma’s (2020) findings also suggest that adequate social support can maintain psychological positivity and counteract negative emotions among athletes. These studies indicate that social support has an ameliorative effect on anxiety generated by external stressors; thus, we propose research hypothesis 1: social support significantly predicts the career anxiety of high-level university soccer players.

Among the internal factors affecting college students’ career anxiety, individual career choice self-efficacy has been found to have a positive impact on career anxiety (Yang, 2024). Employment self-efficacy is derived from Bandura’s theory of self-efficacy, and employment self-efficacy refers to an individual’s belief or confidence in his or her employability (Bandura et al., 2001; Moynihan et al., 2003), Wu et al. (2021) suggests that career choice self-efficacy concerns having confidence in challenging situations, believing in one’s ability to find a suitable job, and having the ability to successfully perform a job role. Shen et al. (2009) argues that the higher an individual’s confidence and evaluation of their own talents in the employment process, the lower their level of career anxiety.

A series of studies support the role played by external social support in the generation and improvement of individual career choice self-efficacy. Through a survey of college graduates, Gao et al. (2020) showed that social support perceptions were closely related to college students’ career anxiety, while individual self-efficacy partially mediated the relational effect. Jiang et al. (2015) showed that social support has a significant positive influence on self-efficacy, i.e., the enhancement of social support can directly promote self-efficacy. This finding emphasizes the positive correlation and mutual facilitation effect between the two, which can mutually help to counteract the generation of negative emotions. Ji (2024) found that perceived social support among adolescents can indirectly act on negative emotions, such as depression, anxiety, and stress, through the mediating role of self-efficacy and noted that low self-efficacy may lead to career choice anxiety. Wang et al.’s (2021) study revealed that people with high self-efficacy tend to experience less anxiety, while Deng and Liu (2025) further showed that individuals with higher self-efficacy can more quickly adapt to environmental changes and develop corresponding coping strategies; conversely, those with lower self-efficacy tend to have difficulty in effectively adjusting and are easily challenged by internal and external factors, which in turn breed deep anxiety. These studies suggest that adequate social support can lead to lower career anxiety, and this positive effect may be because social support can lead to increased career choice self-efficacy. Therefore, we propose research hypothesis 2: career choice self-efficacy has a mediating effect between social support and career anxiety.

For high-level college soccer players, the availability of adequate career planning also affects career anxiety. Kleinknecht and Hefferin consider career planning as an ongoing process of self-assessment and goal setting (Sebastian, 2024). Ding’s (2023) study showed that after an intervention to educate college students about career planning, their Career Anxiety was significantly lower than that of college students who were not intervened. Some studies have shown that immersive career experience and accurate career planning can effectively alleviate Career Anxiety of college students and ensure their mental health (Song, 2025). In addition, Zheng et al. (2016) analyzed how college graduates’ expectations of ideal work units and geographic areas were related to career stress and emotional health, and found that graduates with higher employment expectations significantly experienced more intense stress and career anxiety.

Whereas social support can positively influence an individual’s career planning, Liu (2008) found that social support received by college students not only directly predicts their level of individual career planning, but also indirectly influences individual planning by affecting levels of self-esteem. Several studies have shown that guidance counselors who provide support and assistance to college students with their career planning are effective in alleviating their Career Anxiety, and that this perceived sense of support from a significant other can simultaneously provide other psychological benefits to college students (Mkpoikanke and Maximus, 2025; Ozturk and Doganulku, 2023). Wang et al.’s (2021) study revealed the multifaceted effects of social support on career path design, and found that both aspects of perceived social support and actual benefits of application showed significant correlations with the six elements of career planning (career discovery, self-understanding, goal setting, self-management, feedback from interpersonal interactions, and adjustment and optimization). The results of Huang’s (2023) study showed that social support significantly and positively influences career planning. Zhang and Lin (2025) found a significant negative correlation between career decision-making difficulties and social support. Therefore, existing studies have demonstrated a close relationship between social support and career planning, and have shown that social support can significantly predict career planning, help athletes integrate social resources, develop social network relationships, and largely alleviate soccer players’ career anxiety and resulting stress (Tolentino et al., 2014). Therefore, we propose research hypothesis 3: Career planning has a mediating effect between social support and career anxiety.

**Figure 1:**
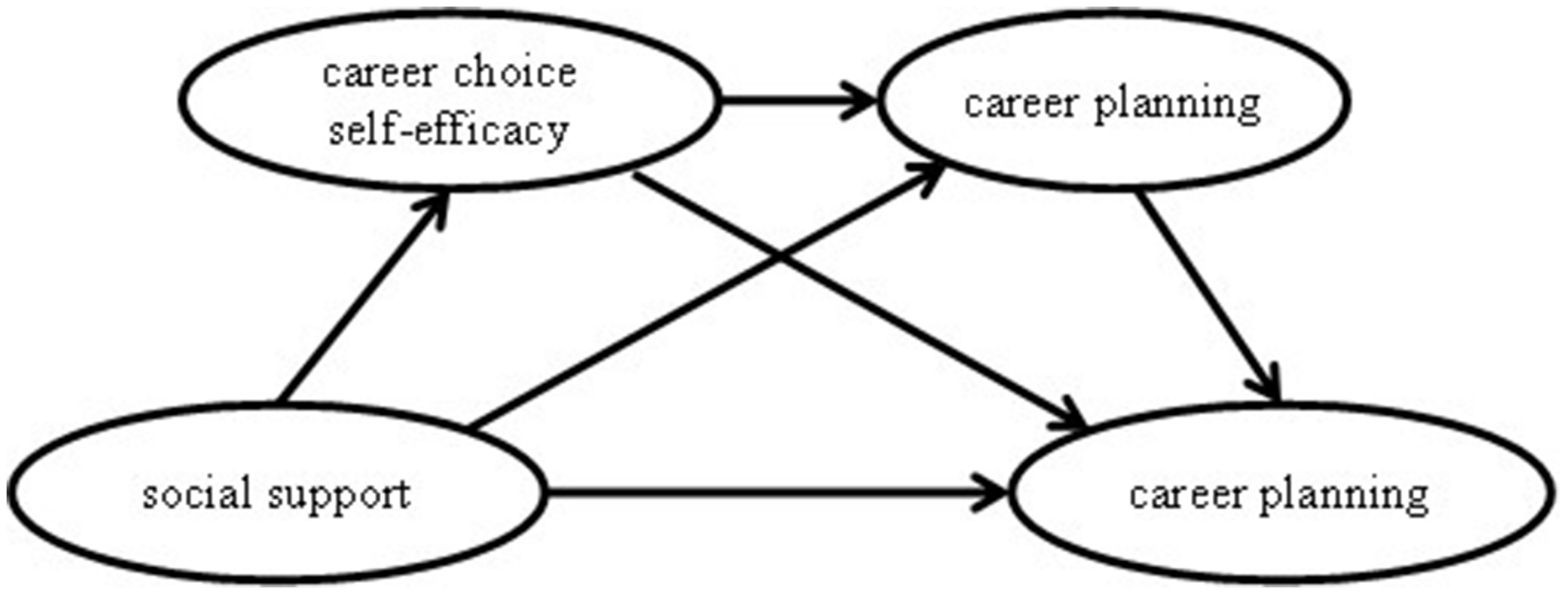
Chain mediation model of factors influencing career anxiety.

The relationship between career choice self-efficacy and career planning is positively correlated. Tolentino et al.’s (2014) view that individuals with higher initiative traits and self-efficacy tend to act positively to plan and adapt their life trajectories is supported by Ebberwein et al.’s (2004) proposition that an individual’s career efficacy plays a positive role in career resilience. This suggests that an individual’s career choice self-efficacy has a positive predictive role in the individual’s career planning process. Gushue et al. found that individuals with a high sense of efficacy demonstrated more accurate career orientation. Not only did they have a clear understanding of themselves, they were also able to set higher career exploration goals and tasks based on their abilities and interests, allowing them to engage in more high-level career development (Gushue et al., 2006). Martin’s (2000) research reveals that college students’ career choice self-efficacy and career planning are closely related, and that students with a high sense of career choice efficacy show a greater ability to adapt to new environments and new jobs. This sense of self-efficacy not only influences individuals’ motivations to choose a career, making them more oriented and determined in their career choices, it also positively affects their career satisfaction. Students with a high sense of career choice efficacy tend to be more satisfied with their career choices and more confident to succeed in their careers (Ebberwein et al., 2004). The positive predictive effect of individual career choice self-efficacy on career planning is supported by several studies. Therefore, synthesizing all of the above studies, we propose hypothesis 4: career choice self-efficacy and career planning have a chain-mediated effect between social support and career anxiety. The chain mediation model is shown in Figure 1.

## 2 Participants and methods

### 2.1 Participants and procedure

The participants were 820 high-level soccer players from three types of universities in China (general education, science and technology, and medicine), the questionnaire distribution period was from November 18, 2024 to January 10, 2025. The questionnaires were administered both online and offline. Written or verbal consent was obtained from the participants before they filled out the questionnaires, for online completion of the questionnaire, participants voluntarily filled it out, which was considered as their informed consent. For offline completion, participants signed an informed consent form on-site. The only inclusion criterion for this study was that students were enrolled in colleges and universities in mainland China. There were no exclusion criteria. All participants were recruited from universities through convenience sampling. A total of 797 (97.19%) valid responses were collected after excluding invalid responses (e.g., monotone responses or careless responses, such as inattention to reverse-worded items and omissions). The participants consisted of 404 males and 393 females, with ages ranging from 18 to 21 years, and with a mean age of 19.55 years (standard deviation = 1.06). These athletes hail from 99 universities across northern China (e.g., Beijing Sport University), central China (e.g., Huazhong Agricultural University), and southern China (e.g., Yunnan Agricultural University). Their athletic classifications include 252 National Level 1 athletes and 545 National Level 2 athletes. The sample size covers a broad geographic range, representing the high-level soccer player population among Chinese university students.

The study was supported by university administrators and physical education teachers. The purpose of the study was presented to administrators, faculty, and participants, and answers were provided anonymously. During the course of the study, participant anonymity was ensured, on-site Q&A was provided, and reverse-worded items were included in the questionnaire. All participants provided written informed consent to participate. The questionnaire took approximately 15 min to complete. The study protocol was approved by the Ethics Committee of Qufu Normal University (ethical approval number is: 2024147) on November 14, 2024. The study was conducted from November 18, 2024 to January 20, 2025. And all study procedures complied with the latest version of the Declaration of Helsinki.

### 2.2 Research method

#### 2.2.1 Social support rating scale

The Social Support Rating Scale (SSRS), developed by Gao and Zhao (2025) was used as an assessment tool. The scale consists of 10 questions including objective support (three questions), subjective support (four questions), and social support utilization (three questions). Questions 1 to 4 and 8 to 10 are rated on a four-point scale, with scores ranging from 1 to 4 in order from not supportive to very supportive. The fifth question concerns social support from family members (loved ones, parents, children, siblings, and other family members), which ranges from no support to full support, with each item assigned a value from 1 to 4. The sixth and seventh questions are scored as zero if the answer was “no source of support.” In instances in which the answer is “no source of support,” zero points are awarded, while, in instances in which there is a source, points are awarded according to the number of sources. The total score range is between 12 and 66, with higher scores indicating a higher level of social support for the individual. The scale has been widely used in research practice in related fields for 30 years. In this study, the Cronbach’s alpha was 0 0.785.

#### 2.2.2 The college graduate career anxiety scale

The level of career anxiety among high-level soccer players was measured using the College Graduates’ Employment Anxiety Scale, developed by Ren (2010). The scale consists of 17 items. A four-point scale is used, with scores from 1 to 4 indicating: no career anxiety at all, somewhat career anxious, mostly career anxious, and completely career anxious. Higher scores indicate a more severe state of career anxiety. In this study, the Cronbach’s alpha of the scale was 0.970.

#### 2.2.3 Career choice self-efficacy scale

The Career Choice Self-Efficacy Scale was developed by Zhang (2008). The questionnaire contains of 22 questions with an internal consistency reliability Cronbach’s coefficient of *α* = 0.908. Examples of test questions include, “Evaluate which career or job you think is of the highest value,” “Clarify your ideal career or job choice,” and “Identify your best career or job area.” The scale is based on a five-point scale from 1 to 5, with higher scores indicating greater self-efficacy in an individual’s career choice. In this study, the Cronbach’s alpha for this scale was 0.964.

#### 2.2.4 Career planning scale for college high-level soccer players

This study used the Career Planning Questionnaire for High-level Athletes in General Colleges and Universities developed by Chen (2021) to measure the participants’ career planning. It consisted of 12 revised questions with topic statements such as “Identify current or future employment trends in a particular occupation or job” and “Judge what you find most valuable in an occupation or job.” The Cronbach’s alpha for the scale was 0.914.

### 2.3 Statistical methods

Descriptive statistics were used to summarize the data characteristics. Pearson correlation analysis was used to investigate associations between study variables. Linear regression models were used to test the effects of predictors and the mediation effect of each mediator. Following multiple mediation effect testing, according to Fang et al. (2014), we tested the mediating effect of career choice self-efficacy and career planning in the relationship between social support and career anxiety. Subsequently, the chain mediation effects of both mediators were tested with serial hierarchical regression analysis (Zhang et al., 2021; Yuan et al., 2024). The above analyses were performed using SPSS 19.0. To further test the internal mechanisms influencing career anxiety, we examined multivariate chained mediation effects within the structural equation modeling (SEM) framework, using AMOS 21.0. A bootstrapping method with 5,000 samples was applied to test the mediation effects. The bias-corrected bootstrap confidence interval was used to test the significance of indirect effects.

## 3 Results

### 3.1 Direct effects of social support, career choice self-efficacy, and career planning on career anxiety

Table 1 shows the Pearson correlations between the study variables. Social support, career choice self-efficacy, career planning, and career anxiety were significantly associated with one another (*p* < 0.01). Career anxiety was significantly and negatively associated with other variables (*p* < 0.01). The results show that the correlation coefficients of career anxiety with social support, career choice self-efficacy, and career planning had high level negative correlations.

**Table 1:** Descriptive statistics and Pearson correlation between study variables (*N* = 797).

Variables	Social support	Career choice self-efficacy	Career planning	Career planning	*M*	*SD*
Social support	1				38.76	6.97
Career choice self-efficacy	0.746**	1			76.49	16.46
Career planning	0.670**	0.748**	1		23.58	7.54
Career anxiety	−0.736**	−0.810**	−0.889**	1	33.59	11.56

*p < 0.05; **p < 0.01; ***p < 0.001.

Table 1 results also indicate high correlation coefficients among variables, necessitating multicollinearity testing. Test results reveal tolerance and variance inflation factors for individual variables as follows: social support (0.407, 2.459), occupational self-efficacy (0.333, 3.001), career planning (0.397, 2.522). Tolerance values for all variables exceed 0.1, and VIF < 5, indicating no severe multicollinearity among the variables.

Table 2 shows the linear regression direct effects of social support, career choice self-efficacy, and career planning on the dependent variable of career anxiety. Social support (F_(1,796)_ = 939.86, *p* < 0.001), career choice self-efficacy (F_(1,796)_ = 1519.21, p < 0 0.001), and career planning (F_(1,796)_ = 2996.37, *p* < 0.001) significantly and negatively predicted career anxiety, explaining 54.2, 65.6, and 79.0% of the variance in career anxiety, respectively. This result supports hypothesis 1.

**Table 2:** Simple direct linear regression on career anxiety.

Model	Career anxiety					
	*B*	*SE*	*β*	*t*	*P*	*R^2^*
Social support	−1.220	0.040	−0.736	−30.657	0.000	0.542***
Career choice self-efficacy	−0.569	0.015	−0.810	−38.977	0.000	0.656***
Career planning	−1.363	0.025	−0.889	−54.739	0.000	0.790***

*p < 0.05; **p < 0.01; ***p < 0.001.

### 3.2 The mediating effect of career choice self-efficacy

Hierarchical regression was used to test the mediating effect of career choice self-efficacy between social support and career anxiety. In the first step, the direct effect of social support on career anxiety was tested. The results showed that social support was a significant negative predictor of career anxiety (F_(1,796)_ = 939.86, *β* = −0.736, R^2^ = 0.542, *p* < 0.001), with Model 1 explaining 54.20% of the variance in variance of career anxiety. Career self-efficacy was added to the model in the second step, with “social support” and “career self-efficacy” as independent effects; these two independent regression effects on career anxiety remained significant (F_(2,796)_ = 906.37, R^2^ = 0.695, *p* < 0.001). Together, the two variables explained 69.5% of the variance in career anxiety, and the value-added contribution of career choice self-efficacy to the regression model was ΔR^2^ = 15.3% (*p* < 0.001). In Model 2, both social support (*β* = −0.296, *p* < 0001) and career choice self-efficacy (β = −0.589, *p* < 0.001) were significant (*p* < 0.001) in the direct prediction of career anxiety, suggesting that career choice self-efficacy acted as a partial mediating variable between social support and career anxiety. This result supports research hypothesis 2.

**Figure 2:**
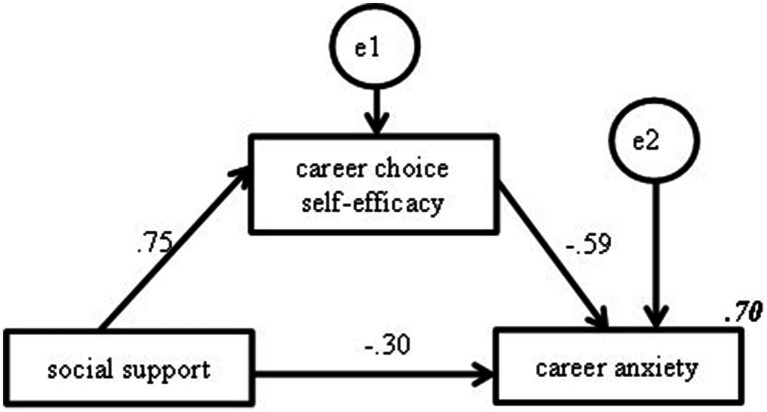
Partial mediation effect model of career choice self-efficacy (*p* < 0.05). Note: The italicized and bolded values in Figure 2 represent the percentage of variance in the dependent variable.

The mediating effect of career choice self-efficacy was further examined using structural equation modeling. The variable relationships are shown in Figure 2, which further supports the results of Table 3. The results of Table 4 indicate that the mediation of career choice self-efficacy accounted for 59.6% of the total effect.

**Table 3:** Hierarchical regression model of social support and career choice self-efficacy on career anxiety.

Model		*B*	*SE*	*β*	*t*	*P*	*F*	*R* ^2^
1	(Constant)	80.886	1.567		51.603	0.000		
	Social support	−1.220	0.040	−0.736	−30.657	0.000	939.86	0.542***
2	(Constant)	84.279	1.290		65.336	0.000		
	Social support	−0.492	0.049	−0.296	−10.075	0.000		
	Career choice self-efficacy	−0.414	0.021	−0.589	−20.014	0.000	906.37	0.695***

*p < 0.05; **p < 0.01; ***p < 0.001.

**Table 4:** Strength of mediating effect of career choice self-efficacy.

Effect	Effect value	Effect strength
Direct effect	−0.30	40.40%
Indirect effect	−0.4425	59.60%
Total effect	−0.7425	100%

### 3.3 The mediating effect of career planning

Stratified regression was used to test the mediating effect of career planning between social support and career anxiety. In the first step, the direct effect of social support on career anxiety was tested. The results showed that social support was a significant negative predictor of career anxiety, with significant regression coefficients and regression models (F_(1,796)_ = 939.86, *β* = −0.736, R^2^ = 0.542, *p* < 0.001), and 54.20% of variance in career anxiety variance was explained by Model 1. In the second step, career planning was added to the model, and the independent variables of “social support” and “career planning” had a significant effect on career anxiety. The regression effect of these independent variables on career anxiety remained significant (F_(2,796)_ = 1883.92, R^2^ = 0.826, *p* < 0.001). Together, the two variables explained 82.6% of the variance in career anxiety, and the value-added contribution of career planning to the regression model was ΔR^2^ = 28.5% (*p* < 0.001). In Model 2, both social support (β = −0.254, *p* < 0.001) and career planning (β = −0.718, *p* < 0.001) were significant in the direct prediction of employment anxiety (*p* < 0.001), suggesting that career planning acted as a partial mediating variable between social support and career anxiety. This result supports research hypothesis 3.

**Figure 3:**
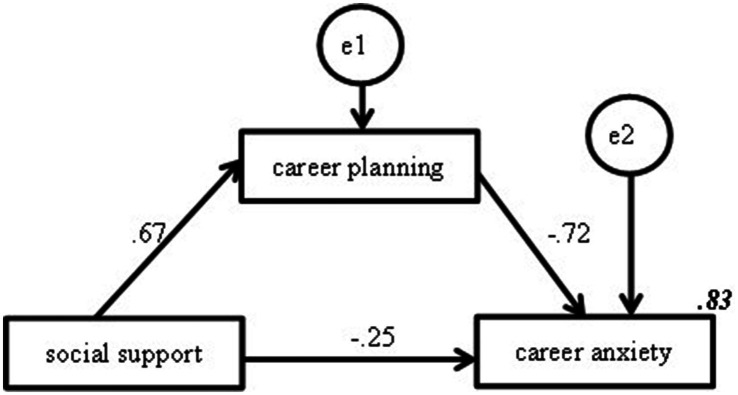
Partial mediation effect model of career planning (*p* < 0.05). Note: The italicized and bolded values in Figure 3 represent the percentage of variance in the dependent variable.

The mediating effect of career planning was further examined using structural equation modeling, and the variable relationships, shown in Figure 3, are consistent with the results of Table 5. The results of Table 6 show that the mediating effect of career planning accounts for 65.87% of the total effect.

**Table 5:** Hierarchical regression model of social support and career planning on career anxiety.

Model		*B*	*SE*	*β*	*t*	*P*	*F*	*R* ^2^
1	(Constant)	80.886	1.567		51.603	0.000		
	Social support	−1.220	0.040	−0.736	−30.657	0.000	939.86	0.542***
2	(Constant)	75.909	0.976		77.738	0.000		
	Social support	−0.422	0.033	−0.254	−12.750	0.000		
	Career planning	−1.101	0.031	−0.718	−36.006	0.000	1883.92	0.826***

*p < 0.05; **p < 0.01; ***p < 0.001.

**Table 6:** Strength of mediating effects of career planning.

Effect	Effect value	Effect strength
Direct effect	−0.25	34.13%
Indirect effect	−0.4824	65.87%
Total effect	−0.7324	100%

### 3.4 Chain-mediated effects of career choice self-efficacy and career planning

**Figure 4:**
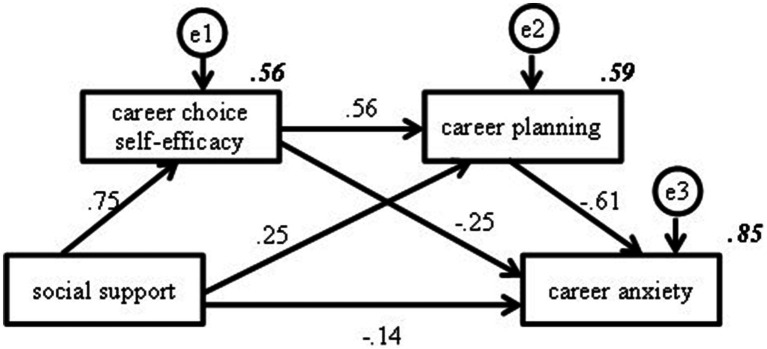
Chain mediation model of career choice self-efficacy and career planning. Note: The italicized and bolded values in Figure 4 represent the percentage of variance in the dependent variable.

The chain-mediated effects of career choice self-efficacy and career planning were tested using structural equation modeling. The variable relationships and path coefficients are shown in Figure 4, in which all predicted paths were significant and the chain mediation model explained 85% of the variance in the dependent variable, career anxiety. The percentage of indirect path effects of the chained mediation model is shown in Table 7, in which the chained mediation effects of career choice self-efficacy and career planning account for 34.80% of the total effect. These results support research hypothesis 4.

**Table 7:** Strength of direct and indirect effects of the chained mediation model.

Effect		Effect value	Effect strength
Direct effect	Social support → Career anxiety	−0.14	19.02%
	Social support → Career choice self-efficacy → Career anxiety	−0.1875	25.47%
Indirect effect	Social support → Career planning → Career Anxiety	−0.1525	20.71%
	Social support → Career choice self-efficacy → Career planning → Career anxiety	−0.2562	34.80%
Total effect		−0.7362	100%

## 4 Discussion

This study explored the influencing factors of employment anxiety and its psychological mechanisms in Chinese university high-level soccer players. The results show that social support significantly negatively predicted employment anxiety. Career choice self-efficacy and employment planning acted as partial mediator variables between social support and employment anxiety, respectively, and that career choice self-efficacy and employment planning had a significant chain mediation role between social support and employment anxiety. The results support our four hypotheses.

### 4.1 Social support and career anxiety

This study found that social support has a positive buffering effect on career anxiety. Over the years, research on the ameliorative effects of social support on mental health, especially negative emotions, has been supported by several studies. Ji (2024) found that individually perceived social support was effective in alleviating negative emotions such as anxiety, depression, and stress. Wang et al.’s (2021) study of college graduates found that social support had a significant negative predictive effect on career anxiety, indicating that adequate social support can effectively alleviate the negative emotions related to employment stress. Yuan et al. (2024) also found that social support significantly negatively predicted college students’ career anxiety, and Zhang and Jenatabadi (2024) showed that social support significantly improved college students’ anxiety in stressful situations. Metts and Craske (2023) examined the relationship between social support and cognitive reappraisal. Social support offers protection from depression and anxiety, possibly through its beneficial effects on cognitive reappraisal. The results suggest that cognitive reappraisal with the influence of social support may be more effective than cognitive reappraisal without social influence, and thus may be a more suitable target for depression and anxiety interventions. A U.S. study showed that multidimensional social support was negatively associated with students’ anxiety and depression in stressful situations (Jamil and Su, 2024). A study by Lindsay et al. (2022) found that interventions involving social support for college students facilitated the recovery of negative emotions such as anxiety in stressful situations. The above studies suggest that social support is positively significant for college students’ mental health and that a strong social support network can empower students with sufficient psychological resources to cope with career anxiety. The results of the present study indicate that social support negatively predicts career anxiety in Chinese college soccer players, suggesting that intervention strategies targeting social support can reduce anxiety and help these students to positively cope with the employment stress they face. The present results are consistent with those of previous studies.

### 4.2 Mediating effects of career choice self-efficacy between social support and career anxiety

Several studies have shown that the most powerful psychological resource that social support can generate for individuals is the ability to improve self-efficacy. Ji (2024) found that social support improves negative emotions such as anxiety through self-efficacy, while Xie (2024) found that the more social support an individual perceives, the higher their self-efficacy, which enables the individual to effectively counteract negative emotions such as depression and anxiety. Zhang and Jenatabadi (2024) found that social support among college students improves their self-efficacy in stressful situations and reduces and relieves stress-induced anxiety. A study by Zhang et al. (2023) found that college students majoring in art perceived social support as a negative predictor of career anxiety, while self-efficacy had a significant mediating effect between the two. A study by Jiang (2024) found that social support for college students negatively predicted career anxiety, while psychological capital partially mediated the relationship between the two, with self-efficacy as a basic component of psychological capital. Zhang et al. (2025) also found that college students’ perceived social support and self-efficacy negatively predicted career anxiety at the same time, and that self-efficacy had a significant mediating effect between social support and career anxiety. Zhang and Zhang (2022) showed that the effect of social support on negative emotions arising from stressful situations (e.g., exercise, employment) was often mediated by individual self-efficacy. Rosa et al. (2022) demonstrated that perceived social support and self-efficacy among Peruvian college students were positive predictors of the students’ ability to cope with stress and negative emotions.

The results of the present study showed that perceived social support for Chinese university high-level soccer players co-influenced their career anxiety through the partial mediation of self-efficacy. This suggests that social support resources not only can directly influence career anxiety, but can indirectly influence career anxiety by increasing career choice self-efficacy. The findings of this research are consistent with those of previous studies.

### 4.3 Mediating effects of career planning between social support and career anxiety

Career planning includes, among other things, an individual’s thinking and decision-making processes, and adaptation to a career. The excessive psychological pressure experienced by college students during the career selection period can produce anxiety that has mental health impacts. Social support resources can help college students to improve their career planning practices and reduce career anxiety. Sun’s (2023) study shows that the career-related social support (including emotional, informational, resource, behavioral, etc.) received by college students during their job search can enable them to engage in better career planning and have stronger career adaptability, thus effectively reducing anxiety due to confusion and uncertainty about the future. Xu (2023) found that the social support received by college students directly and positively predicts the maturity of their career planning, enabling them to adapt to career development situations and to psychologically prepare for employment. Li (2021) found that social support, including substantial material support, emotional encouragement and understanding, clear information, and effective advice, can reduce difficulties in career decision-making, improve career planning abilities, and reduce the career anxiety caused by job search pressure. Zhou et al. (2024) found that social support has a direct effect on college students’ career decision-making and planning and this can effectively reduce employment stress and negative emotions. A study of Korean youth found that social support from significant others, such as parents, had a direct impact on students’ career planning and adjustment, which in turn influenced more positive emotions and employment satisfaction (Hyojung and Jay, 2015), while Hedva et al. (2012) found that insecure attachment had a significant impact on career planning, anxiety, and pessimism associated with career decision-making, and suggested that the support of significant others had a positive impact on career planning and development, and a preventive effect on the development of negative emotions.

The results of this study show that perceived social support among Chinese university high-level soccer players can not only directly influence career anxiety, it can also have an indirect influence through the partially mediated role of career planning.

### 4.4 Chain-mediated effects of career choice self-efficacy and career planning

Jiang found that social support had a positive effect on career choice self-efficacy, and the improvement of self-efficacy had a positive effect on career decision-making. The study also pointed out that these relationships might be influenced by individual personality traits (Jiang, 2022). Wang and Sun (2024) investigated the employment challenges of college students and found that social support had a positive effect on psychological capital when students were job-hunting, that career choice and career decision-making self-efficacy had a chain-mediated effect, and that the enhancement of psychological capital was conducive to positively coping with career anxiety when job-hunting, with increasing stress resistance, and with problem-solving ability. Young and Soo (2018) conducted a study on 853 college students in China and found that the higher perceived social support, the higher the self-efficacy, and that social support and self-efficacy acted simultaneously on college students, helping the students to engage in more employment thinking and planning. Kang and Kang (2015) found that perceived social support increased college students’ career choice self-efficacy, and the higher their self-efficacy, the higher their levels of career planning and decision-making, Young and Soo’s (2018) study also supported the above proposition. Zhou et al., 2024 showed that social support not only has a direct effect on college students’ career decision-making and planning, it also indirectly affects the level of career planning through the mediating effect of career choice self-efficacy, thus reducing career anxiety. The results of a study by Kleine et al. (2023) and Zhao and Shi (2024) on Dutch graduating college students found that career self-efficacy was positively related to career planning and decision-making, and that career-related self-efficacy and career planning were malleable positive cognitive resources that had a chained effect on pre-employment emotional dysregulation.

The results of this study showed that the social support received by Chinese university high-level soccer players enabled them to cope positively with career anxiety, and that their career choice self-efficacy and career planning chain mediated the relationship between social support and career anxiety.

Chinese university high-level soccer players are a special group among college students. Current research on this group mainly focuses on aspects of their athletic ability and physical training (Kang and Kang, 2015), with relatively few studies focusing on their employment prospects and employment-induced negative emotions. This study explored the effects of social support resources on employment anxiety among this group of university students, while further exploring the psychological mechanisms by which social support affects employment anxiety. We found that career choice self-efficacy and career planning have a chain-mediated effect between the two. The above results provide a theoretical reference for intervening in the development of negative emotions, such as career anxiety, among high-level soccer players. The results of this study support the expected hypotheses.

### 4.5 Research limitations

First, the study did not test for gender differences, although other studies have found differences between male and female high level soccer players with regard to range of employment choices. However, this study did not test for gender differences in the study of negative emotions, such as anxiety and restlessness, related to career anxiety.

Second, the research consisted of a cross-sectional survey only and did not include an intervention study. We recommend that future researchers include an intervention study for the theoretical model.

Third, while many factors influence the relationship between social support and career anxiety, this study only explored the role of possible mediating variables. We recommend the future exploration of moderating variables as a meaningful research direction.

Fourth, the sample may exhibit differences in age, gender, years of training, and athletic level. For the sake of research simplicity, this study did not further explore these variables. Subsequent research will continue to deepen the investigation of moderating variables.

Fifth, the timing of the survey coincided with the period of final exams and early job hunting at Chinese universities. The heightened anxiety among college students during this timeframe may also have influenced the results.

## 5 Conclusion

The more social support resources, the higher the level of career choice self-efficacy, the higher the degree of career planning, and the lower the level of career anxiety among high-level soccer players in Chinese universities. Social support indirectly affects career anxiety through the partial mediating role of career choice self-efficacy. Social support indirectly affects career anxiety through the partial mediating role of career planning. Career choice self-efficacy and career planning act as chain mediators between social support and career anxiety, with a chain mediation strength of 34.8%. The direct predictive power of social support on career anxiety was 70%, which increased to 85% after the chain mediation of career choice self-efficacy and career planning, with a contribution of 15% from the chain mediation model. We suggest that interventions into high-level soccer players’ career anxiety should not only provide more social support resources, but should also improve their self-efficacy and career planning ability.

## Data Availability

The original contributions presented in the study are included in the article/supplementary material, further inquiries can be directed to the corresponding author.
